# Junctional sensitivity and atrophy progression rate of geographic atrophy secondary to age‐related macular degeneration

**DOI:** 10.1111/aos.70151

**Published:** 2026-04-18

**Authors:** Alexander Kai Thomsen, Jenni Martinez Villarruel Hinnerskov, Miranda Maung, Aubin Mutschler, SriniVas Sadda, Torben Lykke Sørensen

**Affiliations:** ^1^ Clinical Eye Research Division, Department of Ophthalmology Zealand University Hospital Roskilde Denmark; ^2^ Department of Clinical Medicine University of Copenhagen Copenhagen Denmark; ^3^ Bowdoin College Brunswick Maine USA; ^4^ Department of Ophthalmology Boston Children's Hospital Boston Massachusetts USA; ^5^ Department of Neuroscience Middlebury College Middlebury Vermont USA; ^6^ Department of Ophthalmology Harvard Medical School Boston Massachusetts USA; ^7^ Doheny Eye Institute Los Angeles California USA; ^8^ Department of Ophthalmology, David Geffen School of Medicine University of California – Los Angeles Los Angeles California USA

**Keywords:** age‐related macular degeneration, fundus autofluorescence pattern, geographic atrophy, growth, microperimetry, progression rate, retinal sensitivity

## Abstract

**Purpose:**

To determine the relationship between the baseline junctional retinal sensitivity measured with microperimetry and the atrophy progression rate in eyes with geographic atrophy (GA) secondary to age‐related macular degeneration.

**Methods:**

In this prospective cohort study, patients with GA were examined with fundus autofluorescence (FAF) to determine the atrophy area at baseline and at follow‐up of 13.1 ± 2.5 months in order to determine the atrophy progression rate. Microperimetry and optical coherence tomography (OCT) were evaluated at baseline. Microperimetry was superimposed on the baseline FAF image to determine mean junctional sensitivity. Multivariable linear regression was performed adjusting for age, presence of junctional intraretinal hyperreflective foci (HRF), subretinal drusenoid deposits (SDD) and FAF pattern.

**Results:**

Fifty‐six eyes with GA of 56 patients were included. There was a significant association between baseline junctional sensitivity and the atrophy progression rate in GA eyes (β, −8.84 μm/year/dB; 95% CI, −16.56 to −1.11; *p* = 0.026) tested with simple linear regression. However, when adjusting for age, structural factors and junctional FAF patterns in a multivariable linear regression model, the association between junctional sensitivity and atrophy progression rate was no longer statistically significant (β, −6.40 μm/year/dB; 95% CI, −16.04 to 3.25; *p* = 0.18). The diffuse trickling FAF pattern was independently and significantly associated with progression rate (*p* = 0.019).

**Conclusions:**

Microperimetric junctional sensitivity may have relevance in predicting atrophy progression rate in eyes with GA, although the FAF pattern may be of greater importance. Larger, longitudinal studies are necessary to further elucidate this relationship.

## 1 INTRODUCTION

The late stage of dry age‐related macular degeneration (AMD) is characterized by the development of geographic atrophy (GA). GA is defined as the presence of sharply demarcated lesions characterized by the loss of photoreceptors, retinal pigment epithelium (RPE) and choriocapillaris (Sadda et al., 2018). Atrophic lesions are clearly visualized with fundus autofluorescence (FAF) imaging, with the atrophy appearing dark and in high contrast due to the loss of RPE lipofuscin autofluorescence (Choudhry et al., 2010; Pfau et al., 2023). These atrophic lesions lead to irreversible vision loss, with dense scotomas corresponding to the lesion location, which may be perifoveal or involve the fovea (Pilotto et al., 2013). Perifoveal atrophy affects visual abilities such as reading, driving and seeing in low‐light conditions (Brown et al., 2014; Sunness et al., 2008). Foveal atrophy can cause a severe decline in central vision. However, visual acuity does not correlate well with visual dysfunction in GA, as patients with foveal sparing may retain central vision when measured in a clinical setting yet still experience difficulties with activities of daily living (Csaky et al., 2019). Thus, visual function tests assessing individual points of the macula, such as microperimetry and reading speed, have been shown to be more reliable for grading and monitoring visual dysfunction in GA patients (Sayegh et al., 2014; Wu et al., 2025). Microperimetry measures threshold light sensitivity at multiple points across the macula, mapping their locations, making it useful for correlating retinal structure with function in GA (Cassels et al., 2018; Wong et al., 2017).

Multiple studies have demonstrated a decline in retinal sensitivity over time in patients with GA (Batıoğlu et al., 2014; Biarnés et al., 2015; Holz et al., 2007; Jeong et al., 2014; Schmitz‐Valckenberg et al., 2011). It is also well established that retinal sensitivity is reduced at the junctional zone of GA lesions compared to the retina more distal to the lesion (Hartmann et al., 2011; Querques et al., 2012; Takahashi et al., 2016). Studies evaluating GA lesions with defect‐mapping microperimetry have found that the proportion of missed junctional points could serve as an efficient metric for detecting longitudinal progression of GA extent (Saeed et al., 2025; Wu et al., 2024). Meleth et al. found that these junctional points decreased in sensitivity with time, and the number of scotomatous points increased as the area of the atrophic lesion increased (Meleth et al., 2011). However, the authors did not find a significant association between a change in junctional sensitivity and atrophy progression.

Predicting GA progression is crucial for early intervention and personalized treatment planning, potentially improving visual outcomes for patients. Prognostic factors for progression may reflect differences in the underlying pathophysiology of GA (Niu et al., 2016). Microperimetric retinal sensitivity is associated with GA lesion area, and changes in junctional sensitivity are correlated with the rate of atrophy progression (Chang et al., 2024; Heier et al., 2020). If baseline retinal sensitivity can predict the atrophy progression rate in GA eyes, it could potentially serve as a useful tool for future disease monitoring and treatment strategies. To our knowledge, no previous studies have investigated the predictive value of retinal sensitivity for the atrophy progression rate in GA.

Therefore, we aimed to investigate the association between baseline junctional retinal sensitivity and the atrophy progression rate in eyes with GA.

## 2 METHODS

### 2.1 Study design and participants

In this prospective observational study, patients with GA were enrolled at the Department of Ophthalmology, Zealand University Hospital, Roskilde, Denmark. The study was approved by the Regional Committee of Ethics in Research of the Region of Zealand, Denmark (journal no. SJ‐768) and was performed in adherence to the Declaration of Helsinki. Verbal and written informed consent were obtained from all participants prior to inclusion.

The inclusion criteria were the presence of GA secondary to AMD. Participants were included in a study investigating immunological factors associated with GA; therefore, the exclusion criteria were inflammatory diseases, immunomodulating treatment, active smoking, C‐reactive protein levels >15 mg/L, prior or current neovascular AMD in any eye and previous intravitreal anti‐vascular endothelial growth factor treatment.

Participants underwent pupillary dilation with tropicamide 1% and phenylephrine 2.5% before the assessment of best‐corrected visual acuity (BCVA), microperimetry and multimodal imaging, including colour fundus photography (CFP), spectral‐domain optical coherence tomography (OCT) and FAF imaging. Participants were examined at baseline and at a follow‐up visit after a minimum of 7 months. For each participant, the eye with GA was selected as the study eye; if GA was bilateral, the right eye was chosen (Martinez Villarruel Hinnerskov et al., 2024).

### 2.2 Retinal sensitivity assessment

Retinal sensitivity was evaluated with microperimetry performed using the Nidek MP‐3 (Nidek Technologies, Padua, Italy) after pupil dilation and before other imaging that might otherwise bleach the photoreceptors. The examination was conducted in a dark, quiet room. Study eyes were examined while the contralateral eye was occluded. A stimulus grid assessing the macula consisted of 33 points located at 0°, 2°, 4°, 6°, 8° and 10° from fixation, and the dynamic range of the stimulus brightness was 34 dB. The background illumination was 31.4 abs, and a Goldmann III (radius 62.5 μm, white, 200 ms) stimulus was used with the response threshold determined using a 4–2 staircase strategy. The examination concluded with a CFP to document the position of the stimulation points (Thomsen et al., 2024).

### 2.3 Atrophy progression rate

The FAF and OCT images were acquired using the Heidelberg Spectralis HRA + OCT (Heidelberg Engineering, Heidelberg, Germany). OCT scans were acquired with a 20° × 20° scan field centred on the fovea, consisting of 49 B‐scans, 512 A‐scans per B‐scan and 25 averaged frames in automatic real time. The FAF images were analysed to assess the atrophy area at baseline and follow‐up. These area measurements have been described in detail previously (Martinez Villarruel Hinnerskov et al., 2024). In brief, a semi‐automated MATLAB‐based Image Segmenter (MathWorks, Natick, MA, USA) was used to outline the atrophy and calculate the area. Manual constraints were added to exclude OCT‐verified normal foveal regions, the optic disc and vessel hypoautofluorescence. The atrophy progression rate was determined by subtracting the baseline area from the follow‐up area and dividing this value by the follow‐up time. A square root transformation of the areas was applied to eliminate heteroskedasticity (Feuer et al., 2013).

### 2.4 Structure‐function relationship

The baseline microperimetry CFP with the stimulus spot locations was superimposed onto the baseline FAF using third‐party software (‘Chime Software Ltd. Glass2K. https://chime.tv/products/glass2k.shtml; Accessed February 1, 2024’ n.d.). This process involved rendering the microperimetry CFP transparent and manually adjusting the scale, position and rotation for precise alignment. Retinal vessels served as reference points for accurate superimposition. All images were analysed independently by two graders (M.M. and A.M). A third senior grader (A.K.T.) resolved any cases of disagreement. Graders were blinded to atrophy progression rate data.

Retinal sensitivity points were categorized as junctional, distal or intralesional points. The stimulus points have a diameter of 125 μm (radius of 62.5 μm), so stimulus points whose centres fall just within 62.5 μm of the atrophic region's edge may still stimulate photoreceptors immediately adjacent to the atrophy. Thus, the junctional zone was defined as a band with a width of 500 μm surrounding the atrophic lesion, with an inner margin 62.5 μm internal to the edge of the lesion border and an outer margin 437.5 μm outside of the lesion border (Hariri et al., 2016). Microperimetry points with a centre within this zone were classified as junctional points. The distal zone was defined as the region further than 437.5 μm from the lesion border (Figure 1). In eyes with multifocal lesions, there could be an overlap between the junctional zone surrounding each lesion. The same criteria were applied regarding the distance of 437.5 μm outside the closest lesion (Figure S1). Microperimetry points with a centre within the distal zone were classified as distal points. Points that fell entirely within the GA lesion with a centre greater than 62.5 μm from the lesion edge were classified as intralesional and were not included in further analyses. The combination of junctional and distal points was considered to be extralesional. The distance from the stimulus points to the atrophic lesion border was measured in HEYEX (Heidelberg Engineering, version 1.10.4.0) on the superimposed images using the distance‐measuring tool. The mean baseline junctional, distal and total extralesional sensitivity were compared to the atrophy progression rate.

**FIGURE 1:**
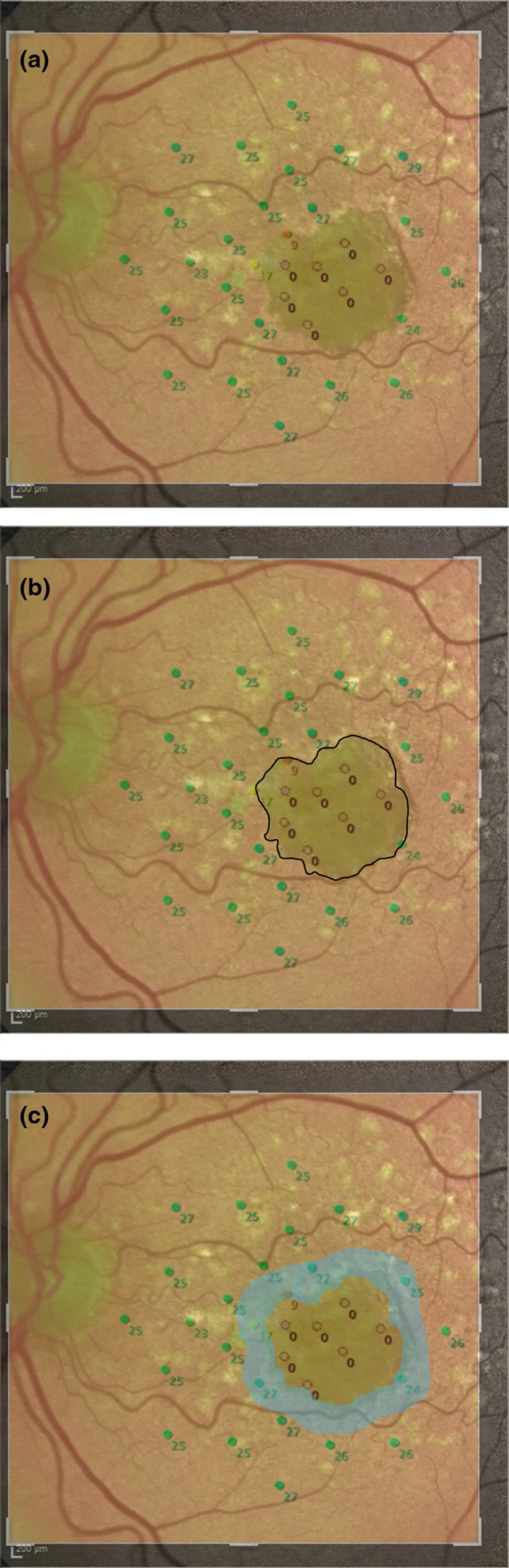
(a) Example of microperimetry colour fundus photograph superimposed on fundus autofluorescence image. (b) Atrophic lesion demarcated. (c) Junctional points are within a 500 μm width band from 62.5 μm inward of the lesion border to 437.5 μm outside of the lesion border. Distal points are more than 437.5 μm from the lesion border.

### 2.5 Statistics

Statistical analysis was conducted using R software version 4.2.3 (R Foundation for Statistical Computing, Vienna, Austria). Continuous normally distributed data is presented as mean and standard deviation. Continuous non‐normally distributed data is presented as median and interquartile range (IQR). Normality was tested with histograms and the Shapiro–Wilk test. A comparison of the mean sensitivity of the junctional and distal points was performed with the Welch Two Sample t‐test. Simple linear regression was performed to assess the correlation between retinal sensitivity and the atrophy progression rate. In this model, the retinal sensitivity was averaged across FAF patterns. Multiple linear regression was also performed to assess the correlation between baseline junctional sensitivity and the atrophy progression rate, adjusting for potential confounders that might affect the progression rate. Adjustments were made for age (Caire et al., 2014), the presence of junctional intraretinal hyperreflective foci (HRF) (Schmidt‐Erfurth et al., 2020), the presence of junctional subretinal drusenoid deposits (SDD) (Marsiglia et al., 2013) and the junctional FAF pattern (Holz et al., 2007) as these factors have been reported to affect the atrophy progression rate in GA patients. HRF and SDD were evaluated on OCT in the junctional zone, from the lesion border to 437.5 μm outside the lesion border. Junctional FAF patterns were classified as none, focal, patchy, banded, diffuse non‐trickling and diffuse trickling (Fleckenstein et al., 2018). An additional multiple linear regression analysis was performed to assess the correlation between baseline junctional sensitivity and the atrophy progression rate, adjusting for lesion focality and baseline atrophy area, rather than FAF pattern. Lesion focality and baseline junctional area have been shown to be important predictors of atrophy progression rate, but also have high collinearity with FAF pattern (Biarnés et al., 2015; Shen et al., 2021). Lesion focality was classified as unifocal and multifocal.

As only one eye per patient was included and only one value of retinal sensitivity was evaluated per analysis, a mixed effects model adjusting for related data was not necessary. The results are presented as a beta coefficient (β) and a 95% confidence interval (95% CI). A *p* value <0.05 was interpreted as statistically significant.

## 3 RESULTS

Fifty‐six eyes with GA from 56 patients were included in this study, of which 33 (58.9%) were female. The mean baseline age of the study population was 78.8 (SD 7.3) years and BCVA was 59.2 (SD 19.0) EDTRS letters. The mean follow‐up period to determine the atrophy progression rate was 13.1 months (SD 2.5). The median number of junctional, distal and total extralesional points was 6.5 (IQR, 4.4), 22.0 (9.0) and 29.0 (IQR, 6.8), respectively. Details on structural and functional factors can be found in Table 1. Mean junctional sensitivity was significantly lower than mean distal sensitivity (mean difference −7.55 dB; 95% CI, −9.53 to −5.57; *p* < 0.001).

**TABLE 1:** Microperimetric retinal sensitivity and structural measures of patients.

Baseline junctional sensitivity (dB), mean (SD)	13.14 (5.87)
Baseline distal sensitivity (dB), mean (SD)	20.69 (4.45)
Baseline total extralesional sensitivity (dB), mean (SD)	18.57 (5.04)
Baseline atrophy area (mm^2^), median (IQR)	4.46 (10.28)
Progression rate (μm/year), mean (SD)^a^	294.96 (174.17)
Junctional HRF presence, *n* (%)	30 (53.6)
Junctional SDD presence, *n* (%)	11 (19.6)
Lesion focality	
Unifocal, *n* (%)	29 (52)
Multifocal, *n* (%)	27 (48)
FAF pattern, *n* (%)	
None	7 (12,5)
Banded	7 (12,5)
Focal	3 (5,4)
Patchy	6 (10,7)
Diffuse non‐trickling	24 (42,9)
Diffuse trickling	9 (16,1)

Abbreviations: HRF, intraretinal hyperreflective foci; FAF, fundus autofluorescence; SDD, subretinal drusenoid deposits.

^a^ Square root transformed.

Simple linear regression revealed a significant inverse association between baseline mean junctional sensitivity and the atrophy progression rate (β −8.84 μm/year/dB; 95% CI, −16.56 to −1.11; *p* = 0.026), indicating faster progression with lower sensitivity. There was no significant association between the mean distal or total extralesional sensitivity and the atrophy progression rate. Multiple linear regression was used to assess the association between junctional sensitivity and the atrophy progression rate, adjusting for potential confounders such as age, baseline junctional HRF presence, baseline junctional SDD presence and FAF pattern. This analysis showed that junctional sensitivity remained negatively associated with the atrophy progression rate; however, this was no longer statistically significant (β, −6.40 μm/year/dB; 95% CI, −16.04 to 3.25; *p* = 0.18) (Table 2).

**TABLE 2:** Association between atrophy progression rate and baseline microperimetric retinal sensitivity.

	Beta coefficient (95% CI)	*p* value
Simple linear regression (μm/year)		
Mean junctional sensitivity (dB)	−8.84 (−16.56 to −1.11)	**0.026**
Mean distal sensitivity (dB)	−3.21 (−13.39 to 6.98)	0.53
Mean total extralesional sensitivity (dB)	−5.31 (−11.75 to 5.94)	0.25
Multivariable linear regression^a^ (μm/year)		
Mean junctional sensitivity (dB)	−6.40 (−16.04 to 3.25)	0.18
Junctional intraretinal hyperreflective foci	−4.26 (−97.06 to 96.03)	0.93
Junctional subretinal drusenoid deposits	−45.40 (−158.66 to 83.64)	0.93
FAF pattern		
None	Ref.	
Banded	98.58 (−88.84 to 285.99)	0.30
Focal	133.00 (−113.85 to 379.85)	0.28
Patchy	80.03 (−114.71 to 274.77)	0.41
Diffuse non‐trickling	114.96 (−4.32 to 294.24)	0.057
Diffuse trickling	226.51 (39.30 to 413.71)	**0.019**

*Note*: Bold values indicate statistical significance (*p* < 0.05).

Abbreviation: CI, confidence interval.

^a^ Multiple regression adjusted for age, presence of junctional intraretinal hyperreflective foci, subretinal drusenoid deposits and fundus autofluorescence (FAF) pattern.

The multiple linear regression model adjusting for age, baseline junctional HRF presence, baseline junctional SDD presence, lesion focality and baseline atrophy area did not show a significant association between junctional sensitivity and atrophy progression rate (−7.89 μm/year/dB; 95% CI, −18.15 to 2.38; *p* = 0.12) (Table S1).

Age and the presence of junctional HRF and SDD were not significantly associated with the atrophy progression rate when tested with simple linear regression, nor were these factors associated with the atrophy progression rate in the multiple linear regression model. Junctional FAF pattern was associated with the atrophy progression rate. The diffuse non‐trickling and diffuse trickling patterns were significantly associated with the progression rate in the simple linear regression (*p* = 0.026 and *p* = 0.0018, respectively) (Table 3). In the multiple linear regression model, only the diffuse trickling FAF pattern was significantly associated with the atrophy progression rate (*p* = 0.019) (Table 2).

**TABLE 3:** Linear regression analysis, evaluating the association between atrophy progression rate and baseline fundus autofluorescence (FAF) pattern adjusted for age.

FAF pattern	Beta coefficient (95% CI) (μm/year)	*p* value
None	Ref.	
Banded	153.67 (−56.31 to 277.82)	0.18
Focal	79.73 (−146.94 to 306.40)	0.48
Patchy	90.03 (−92.72 to 272.78)	0.33
Diffuse non‐trickling	160.73 (19.63 to 301.83)	**0.026**
Diffuse trickling	271.20 (105.67 to 436.74)	**0.0018**

*Note*: Bold values indicate statistical significance (*p* < 0.05).

Abbreviation: CI, confidence interval.

Of note, the mean junctional retinal sensitivity was significantly lower in eyes with diffuse trickling junctional FAF compared to eyes without (mean difference, −5.27 dB, 95% CI, −9.21 to −1.34; *p* = 0.0096).

Because of these results, we also analysed the association between baseline mean junctional sensitivity and atrophy progression rate stratified by FAF pattern (Table S2). This analysis revealed a significant inverse association in eyes with a diffuse trickling pattern (β, −6.31 μm/year/dB; 95% CI, −42.96 to −1.28; *p* = 0.041). No statistically significant associations were found between mean junctional sensitivity and atrophy progression rate in eyes with other FAF patterns.

We also found an association between lesion focality and atrophy progression rate using a simple linear regression model. Multifocal lesions progressed significantly faster than unifocal lesions (mean difference, 107.06 μm/year; 95% CI, 17.46 to 196.67; *p* = 0.020).

## 4 DISCUSSION

This prospective observational study aimed to analyse the association between baseline retinal sensitivity and the atrophy progression rate in patients with GA. In our cohort, we found a mean square root progression rate of 294.96 μm/year, which is similar to previous studies (Domalpally et al., 2016; Yehoshua et al., 2011; Yehoshua et al., 2014). We also found that junctional sensitivity was significantly lower than more distal sensitivity. Atrophic lesions in AMD eyes grow at their margins/borders, enlarging the area of the irreversibly damaged retina and thus the scotomas (Fleckenstein et al., 2021). It is evident that the non‐atrophic junctional retina is damaged with structural degenerative changes in this area, manifesting as hyperautofluorescence on FAF (Brar et al., 2009), irregular RPE elevations (Moussa et al., 2013), splitting of the RPE‐Bruch's membrane complex band (Moussa et al., 2013) and increased inner nuclear layer thickness (Ebneter et al., 2016). Hyperautofluorescence on FAF (Pilotto et al., 2013), irregular RPE elevations (Wu et al., 2014) and increased inner nuclear layer thickness (Mokrane et al., 2021), in particular, have been shown to be associated with decreased sensitivity. Pre‐atrophic degenerative changes have been described as nascent GA, which are reliable predictors of where the atrophic lesion will form (Wu et al., 2015). The significant decrease in junctional sensitivity compared to more distal sensitivity aligns with the presence of the most pronounced degenerative structural changes in the junctional zone. Multiple previous studies also found junctional sensitivity to be decreased compared to distal sensitivity (Hartmann et al., 2011; Querques et al., 2012; Takahashi et al., 2016).

To our knowledge, this is the first study to investigate the association between baseline microperimetric retinal sensitivity and the atrophy progression rate in GA. We found that decreased mean baseline junctional sensitivity was significantly associated with an increased progression rate on univariable linear regression. These results might not be surprising, as multiple structural changes predictive of GA enlargement are observed in the junctional zone, and these structural changes can be associated with decreased retinal sensitivity. The decreased junctional sensitivity was, however, no longer statistically significant after adjusting for the presence of structural risk factors such as the HRF and SDD in the junctional zone, lesion focality, baseline atrophy area and in particular the junctional FAF pattern. A diffuse trickling FAF pattern was independently and significantly associated with atrophy progression rate, suggesting that this pattern is the most important factor for predicting progression rate. However, it should be noted that eyes with the diffuse trickling pattern also featured a lower mean junctional retinal sensitivity. When stratifying by FAF pattern, we found that decreased mean baseline junctional sensitivity was significantly associated with an increased progression rate in eyes with a diffuse trickling pattern adjusted for age. Microperimetry might thus be most useful in predicting progression rate in eyes with a diffuse trickling atrophy pattern. The wide confidence interval observed for the patchy atrophy subtype indicates substantial uncertainty in the estimated structural‐functional relationship and limits the clinical interpretability of subgroup‐specific predictive estimates.

Hariri et al. found that microperimetry could detect a decrease in sensitivity over time at the junctional zone (Hariri et al., 2016). Previous studies have also shown that baseline choriocapillaris flow impairments in the junctional zone predict the atrophy progression rate in GA (Nassisi et al., 2019; You et al., 2021). This flow impairment might in turn cause the overlying RPE to be impaired and vulnerable to atrophic changes (Nassisi et al., 2019). It has also been found that choriocapillaris flow impairments could be detected before the development of nascent GA (Greig et al., 2024). In addition, photoreceptor injury manifesting as disruption or loss of the ellipsoid zone (EZ) has been shown to surround GA lesions (Birner et al., 2025; Sarici et al., 2022), and EZ and RPE loss are associated with decreased retinal sensitivity (Birner et al., 2026). The reduced photosensitivity noted in our cohort may reflect such photoreceptor abnormalities.

Our results align with previous studies that find the diffuse trickling FAF pattern to be associated with significantly increased atrophy progression rate (Fleckenstein et al., 2018; Holz et al., 2007). The mechanistic explanation for the correlation between FAF pattern and progression rate is largely unknown; however, the photoreceptor‐RPE‐ratio has been suggested to be a structural alteration associated with GA progression and found to be associated with FAF pattern (Egger et al., 2025). Likewise, our results support the well‐established association between faster GA progression rate and multifocal lesions compared to unifocal lesions (Shen et al., 2020). Larger lesion perimeter and number, which are associated with focality, were also found to strongly correlate with GA area growth in a previous study (Shen et al., 2021).

Various other structural factors that increase the risk of developing GA have been shown to decrease retinal sensitivity. This includes the degenerative changes: increased drusen volume, the presence of HRF and the presence of SDD (Ouyang et al., 2013). A thin double‐layer sign indicative of thick basal laminar deposit has also been shown to be associated with decreased retinal sensitivity in intermediate AMD patients (Thomsen et al., 2024). This should be distinguished from a thick double‐layer sign that has been associated with nonexudative macular neovascularization, and researchers have speculated that this might be a rescue mechanism for an impaired retina (Fleckenstein et al., 2021; Thomsen et al., 2024). Furthermore, fellow eye characteristics have been shown to be associated with GA development. Patients with GA or macular neovascularization in one eye are more likely to develop GA in the fellow eye, which might be a consequence of the advanced disease stage of both eyes (Fleckenstein et al., 2011; Lindblad et al., 2009). Single nucleotide polymorphisms in the CFH, CFI, CFB and C3 genes have also been suggested to be associated with GA progression (Fleckenstein et al., 2018), as well as systemic inflammatory changes (Krogh Nielsen et al., 2020; Krogh Nielsen, Subhi, Molbech, Falk, Nissen, & Sørensen, 2019; Krogh Nielsen, Subhi, Molbech, Falk, Singh, et al., 2019; Martinez Villarruel Hinnerskov et al., 2024; Riley‐Gillis et al., 2023).

Notably, we did not find a significant association between distal or total extralesional sensitivity and the atrophy progression rate. As the retina distal from the lesion may demonstrate less severe degenerative changes, it may be expected that retinal sensitivity would be better in this region and perhaps less predictive of the progression rate. A previous study did show that eyes with GA had decreased sensitivity in all extralesional regions compared to eyes with early and intermediate AMD. A correlation between a non‐atrophic retina and retinal sensitivity is thus dependent on the AMD stage, with GA eyes showing an overall increase in degenerative progression within the macula (Hariri et al., 2016). Furthermore, prolonged FAF lifetime, which is an indicator of RPE deterioration, has been found to be significantly longer in intermediate AMD eyes that develop GA compared to eyes that remain intermediate (Hammer & Meller, 2023).

Microperimetry plays an increasingly significant role in evaluating GA progression and is widely utilized in both observational and interventional clinical studies (Lad et al., 2024). Accurate prediction of disease progression can facilitate improved monitoring, timely therapeutic interventions and more accurate prognostication for patients. Our findings highlight the prognostic value of microperimetric junctional sensitivity as a predictor of atrophy progression in GA eyes, although FAF pattern might be the most important factor. Future strategies for predicting GA progression may benefit from machine learning approaches, as Ansari et al. demonstrated that such models can reliably predict retinal sensitivity from structural information (Ansari et al., 2025).

Limitations of this study include the relatively few microperimetry points used to assess retinal sensitivity. An important limitation is the relatively low number of junctional points, which reduces the statistical power of the study. Utilizing a denser grid could provide further details and more precise estimates of junctional sensitivity. Including perilesional zones of different widths surrounding the atrophy in future studies might offer a more granular assessment of the contribution of specific segments of the perilesional region to disease progression (Wu et al., 2024). Patients were only tested once per eye with a 4–2 staircase strategy, which could potentially have underestimated losses in visual sensitivity (Wu et al., 2023).

Larger atrophic lesions would also have more junctional zone points, which could potentially skew the results. We adjusted for structural factors, such as HRF and SDD in the junctional zone evident on OCT; however, adjusting for other junctional factors such as the status of outer photoreceptor bands, would provide deeper insights into the role of retinal sensitivity in the atrophy progression rate of GA. Extensive macular atrophy with pseudodrusen‐like appearance has been described as a phenotype associated with rapidly progressive atrophy (Hamel et al., 2009). This phenotype was not explicitly investigated, which is a limitation of this study, but somewhat accounted for by classifying the FAF patterns according to the system suggested by Fleckenstein et al. and adjusting for junctional SDDs (Fleckenstein et al., 2018). This study was also limited by not adjusting for eye‐level SDD, which has been shown to locally affect retinal sensitivity (Kumar et al., 2022).

In conclusion, this study found that decreased junctional sensitivity assessed with microperimetry was associated with the atrophy progression rate in eyes with GA. This association was, however, not statistically significant when adjusting for the junctional FAF pattern, HRF and SDD. When stratifying for FAF pattern, it seems that an association between junctional sensitivity and diffuse trickling FAF pattern was present. Further research incorporating denser sensitivity grids and accounting for additional structural risk factors is necessary to gain a more comprehensive understanding of the relationship between retinal function and atrophy progression in GA patients.

## FUNDING INFORMATION

Financial support was provided by the Velux Foundation (00024226, Søborg, Denmark), Region Zealand PhD Grant (R29‐A1337, Sorø, Denmark), Fight for Sight Denmark (Øjenforeningen), Synoptik Fonden, Dagmar Marshalls Fond and Grosserer L.F. Foghts Fond. The funding organizations had no role in the design or conduct of this research.

## CONFLICT OF INTEREST STATEMENT

SV is a consultant for 4DMT, AbbVie, Alexion, Allergan Inc., Alnylam Pharmaceuticals, Amgen Inc., Apellis Pharmaceuticals, Inc., Astellas, Bayer Healthcare Pharmaceuticals, Biogen MA Inc., Boehringer Ingelheim, Carl Zeiss Meditec, Catalyst Pharmaceuticals Inc., Centervue Inc., Genentech, Gyroscope Therapeutics, Heidelberg Engineering, Hoffmann‐La Roche, Ltd., Iveric Bio, Janssen Pharmaceuticals Inc., Nanoscope, Notal Vision Inc., Novartis Pharma AG, Optos Inc., Oxurion/Thrombogenics, Oyster Point Pharma, Regeneron Pharmaceuticals Inc., Samsung Bioepis, Topcon Medical Systems Inc. Recipient of Carl Zeiss Meditec, Heidelberg Engineering, Nidek Incorporated, Novartis Pharma AG and Topcon Medical Systems Inc. SV received financial support from Carl Zeiss Meditec, Heidelberg Engineering, Optos Inc., Nidek, Topcon and Centervue. AKT, MM, AM, JMVH and TLS have no conflicts of interest to declare.

## ETHICS STATEMENT

All procedures performed in studies involving human participants were in accordance with the ethical standards of the Regional Committee of Ethics in Research of the Region of Zealand, Denmark (journal no: SJ‐768) and with the 1964 Helsinki Declaration and its later amendments.

## INFORMED CONSENT

Informed consent was obtained from all individual participants included in the study.

## Supporting information


Figure S1.

Table S1.

Table S2.

## References

[aos70151-bib-0001] Ansari, G. , Schärer, N. , Pfau, K. , Valmaggia, P. , Gabrani, C. , Zuche, H. et al. (2025) Evaluating the progression of retinal sensitivity loss in geographic atrophy using machine‐learning‐based structure‐function correlation (OMEGA 2). Investigative Ophthalmology & Visual Science, 66, 34. 10.1167/iovs.66.11.34 PMC12366863 40801672

[aos70151-bib-0002] Batıoğlu, F. , Gedik Oğuz, Y. , Demirel, S. & Ozmert, E. (2014) Geographic atrophy progression in eyes with age‐related macular degeneration: role of fundus autofluorescence patterns, fellow eye and baseline atrophy area. Ophthalmic Research, 52, 53–59. 10.1159/000361077 24993093

[aos70151-bib-0003] Biarnés, M. , Arias, L. , Alonso, J. , Garcia, M. , Hijano, M. , Rodríguez, A. et al. (2015) Increased fundus autofluorescence and progression of geographic atrophy secondary to age‐related macular degeneration: the GAIN study. American Journal of Ophthalmology, 160, 345–353. 10.1016/j.ajo.2015.05.009 25982972

[aos70151-bib-0004] Birner, K. , Mai, J. , Boryshchuk, D. , Frommlet, F. , Enzendorfer, M.L. , Schürer‐Waldheim, S. et al. (2026) Exploring trial endpoints in geographic atrophy based on localized functional changes in Microperimetry and AI‐quantified OCT biomarkers. Investigative Ophthalmology & Visual Science, 67, 22. 10.1167/iovs.67.1.22 PMC12805968 41533918

[aos70151-bib-0005] Birner, K. , Reiter, G.S. , Steiner, I. , Zarghami, A. , Sadeghipour, A. , Schürer‐Waldheim, S. et al. (2025) Structure‐function correlation of deep‐learning quantified ellipsoid zone and retinal pigment epithelium loss and Microperimetry in geographic atrophy. Investigative Ophthalmology & Visual Science, 66, 26. 10.1167/iovs.66.3.26 PMC11918028 40067294

[aos70151-bib-0006] Brar, M. , Kozak, I. , Cheng, L. , Bartsch, D.U. , Yuson, R. , Nigam, N. et al. (2009) Correlation between spectral‐domain optical coherence tomography and fundus autofluorescence at the margins of geographic atrophy. American Journal of Ophthalmology, 148, 439–444. 10.1016/j.ajo.2009.04.022 PMC2731819 19541290

[aos70151-bib-0007] Brown, J.C. , Goldstein, J.E. , Chan, T.L. , Massof, R. , Ramulu, P. & Low Vision Research Network Study Group . (2014) Characterizing functional complaints in patients seeking outpatient low‐vision services in the United States. Ophthalmology, 121, 1655–1662. 10.1016/j.ophtha.2014.02.030 PMC6746569 24768243

[aos70151-bib-0008] Caire, J. , Recalde, S. , Velazquez‐Villoria, A. , Garcia‐Garcia, L. , Reiter, N. , Anter, J. et al. (2014) Growth of geographic atrophy on fundus autofluorescence and polymorphisms of CFH, CFB, C3, FHR1‐3, and ARMS2 in age‐related macular degeneration. JAMA Ophthalmology, 132, 528–534. 10.1001/jamaophthalmol.2013.8175 24557084

[aos70151-bib-0009] Cassels, N.K. , Wild, J.M. , Margrain, T.H. , Chong, V. & Acton, J.H. (2018) The use of microperimetry in assessing visual function in age‐related macular degeneration. Survey of Ophthalmology, 63, 40–55. 10.1016/j.survophthal.2017.05.007 28579549

[aos70151-bib-0010] Chang, D.S. , Callaway, N.F. , Steffen, V. , Csaky, K. , Guymer, R.H. , Birch, D.G. et al. (2024) Macular sensitivity endpoints in geographic atrophy. Ophthalmology Science, 4, 100351. 10.1016/j.xops.2023.100351 PMC10587617 37869030

[aos70151-bib-0011] Chime Software Ltd . (n.d.) Glass2K. https://chime.tv/products/glass2k.shtml

[aos70151-bib-0012] Choudhry, N. , Giani, A. & Miller, J.W. (2010) Fundus autofluorescence in geographic atrophy: a review. Seminars in Ophthalmology, 25, 206–213. 10.3109/08820538.2010.518121 21091001

[aos70151-bib-0013] Csaky, K.G. , Patel, P.J. , Sepah, Y.J. , Birch, D.G. , do, D.V. , Ip, M.S. et al. (2019) Microperimetry for geographic atrophy secondary to age‐related macular degeneration. Survey of Ophthalmology, 64, 353–364. 10.1016/j.survophthal.2019.01.014 PMC6532786 30703401

[aos70151-bib-0014] Domalpally, A. , Danis, R. , Agrón, E. , Blodi, B. , Clemons, T. , Chew, E. et al. (2016) Evaluation of geographic atrophy from color photographs and fundus autofluorescence images: age‐related eye disease study 2 report number 11. Ophthalmology, 123, 2401–2407. 10.1016/j.ophtha.2016.06.025 PMC5077673 27448832

[aos70151-bib-0015] Ebneter, A. , Jaggi, D. , Abegg, M. , Wolf, S. & Zinkernagel, M.S. (2016) Relationship between presumptive inner nuclear layer thickness and geographic atrophy progression in age‐related macular degeneration. Investigative Ophthalmology & Visual Science, 57, OCT299‐306. 10.1167/iovs.15-18865 27409486

[aos70151-bib-0016] Egger, D. , Doll, B. , Gonzalez, C. , Ahmadzai, P. , Heger, K.A. , Kreid, B. et al. (2025) Photoreceptor‐ rpe loss ratio and fundus autofluorescence patterns as predictive factors for lesion progression in geographic atrophy. Acta Ophthalmologica, 103, e204–e212. 10.1111/aos.17431 39692064

[aos70151-bib-0017] Feuer, W.J. , Yehoshua, Z. , Gregori, G. , Penha, F.M. , Chew, E.Y. , Ferris, F.L. et al. (2013) Square root transformation of geographic atrophy area measurements to eliminate dependence of growth rates on baseline lesion measurements: a reanalysis of age‐related eye disease study report no. 26. JAMA Ophthalmology, 131, 110–111. 10.1001/jamaophthalmol.2013.572 PMC11551521 23307222

[aos70151-bib-0018] Fleckenstein, M. , Keenan, T.D.L. , Guymer, R.H. , Chakravarthy, U. , Schmitz‐Valckenberg, S. , Klaver, C.C. et al. (2021) Age‐related macular degeneration. Nature Reviews. Disease Primers, 7, 31. 10.1038/s41572-021-00265-2 PMC12878645 33958600

[aos70151-bib-0019] Fleckenstein, M. , Mitchell, P. , Freund, K.B. , Sadda, S. , Holz, F.G. , Brittain, C. et al. (2018) The progression of geographic atrophy secondary to age‐related macular degeneration. Ophthalmology, 125, 369–390. 10.1016/j.ophtha.2017.08.038 29110945

[aos70151-bib-0020] Fleckenstein, M. , Schmitz‐Valckenberg, S. , Adrion, C. , Visvalingam, S. , Göbel, A.P. , Mössner, A. et al. (2011) Progression of age‐related geographic atrophy: role of the fellow eye. Investigative Ophthalmology & Visual Science, 52, 6552–6557. 10.1167/iovs.11-7298 21757586

[aos70151-bib-0021] Greig, E.C. , Moult, E.M. , Despotovic, I.N. , Hodgson, L.A.B. , Pramil, V. , Fujimoto, J.G. et al. (2024) Assessment of Choriocapillaris flow prior to nascent geographic atrophy development using optical coherence tomography angiography. Investigative Ophthalmology & Visual Science, 65, 33. 10.1167/iovs.65.1.33 PMC10807498 38236187

[aos70151-bib-0022] Hamel, C.P. , Meunier, I. , Arndt, C. , Salah, S.B. , Lopez, S. , Bazalgette, C. et al. (2009) Extensive macular atrophy with Pseudodrusen‐like appearance: a new clinical entity. American Journal of Ophthalmology, 147, 609–620. 10.1016/j.ajo.2008.10.022 19181301

[aos70151-bib-0023] Hammer, M. & Meller, D. (2023) Fundus autofluorescence lifetime might predict progression of amd to geographic atrophy, but not to choroidal neovascularization. Acta Ophthalmologica, 101, 903–904. 10.1111/aos.15728 37337863

[aos70151-bib-0024] Hariri, A.H. , Tepelus, T.C. , Akil, H. , Nittala, M.G. & Sadda, S.R. (2016) Retinal sensitivity at the junctional zone of eyes with geographic atrophy due to age‐related macular degeneration. American Journal of Ophthalmology, 168, 122–128. 10.1016/j.ajo.2016.05.007 27189929

[aos70151-bib-0025] Hartmann, K.I. , Bartsch, D.‐U.G. , Cheng, L. , Kim, J.S. , Gomez, M.L. , Klein, H. et al. (2011) Scanning laser ophthalmoscope imaging stabilized microperimetry in dry age‐related macular degeneration. Retina, 31, 1323–1331. 10.1097/IAE.0b013e31820a6850 21540764

[aos70151-bib-0026] Heier, J.S. , Pieramici, D. , Chakravarthy, U. , Patel, S.S. , Gupta, S. , Lotery, A. et al. (2020) Visual function decline resulting from geographic atrophy. Ophthalmology Retina, 4, 673–688. 10.1016/j.oret.2020.01.019 32199866

[aos70151-bib-0027] Holz, F.G. , Bindewald‐Wittich, A. , Fleckenstein, M. , Dreyhaupt, J. , Scholl, H.P.N. , Schmitz‐Valckenberg, S. et al. (2007) Progression of geographic atrophy and impact of fundus autofluorescence patterns in age‐related macular degeneration. American Journal of Ophthalmology, 143, 463–472. 10.1016/j.ajo.2006.11.041 17239336

[aos70151-bib-0028] Jeong, Y.J. , Hong, I.H. , Chung, J.K. , Kim, K.L. , Kim, H.K. & Park, S.P. (2014) Predictors for the progression of geographic atrophy in patients with age‐related macular degeneration: fundus autofluorescence study with modified fundus camera. Eye, 28, 209–218. 10.1038/eye.2013.275 PMC3930277 24458203

[aos70151-bib-0029] Krogh Nielsen, M. , Subhi, Y. , Molbech, C.R. , Falk, M.K. , Nissen, M.H. & Sørensen, T.L. (2019) Systemic levels of Interleukin‐6 correlate with progression rate of geographic atrophy secondary to age‐related macular degeneration. Investigative Ophthalmology & Visual Science, 60, 202–208. 10.1167/iovs.18-25878 30644965

[aos70151-bib-0030] Krogh Nielsen, M. , Subhi, Y. , Molbech, C.R. , Falk, M.K. , Nissen, M.H. & Sørensen, T.L. (2020) Chemokine profile and the alterations in CCR5‐CCL5 Axis in geographic atrophy secondary to age‐related macular degeneration. Investigative Ophthalmology & Visual Science, 61, 28. 10.1167/iovs.61.4.28 PMC7401724 32324857

[aos70151-bib-0031] Krogh Nielsen, M. , Subhi, Y. , Molbech, C.R. , Falk, M.K. , Singh, A. , Nissen, M.H. et al. (2019) Patients with a fast progression profile in geographic atrophy have increased CD200 expression on circulating monocytes: CD200 expression in geographic atrophy. Clinical & Experimental Ophthalmology, 47, 69–78. 10.1111/ceo.13362 30047199

[aos70151-bib-0032] Kumar, H. , Guymer, R.H. , Hodgson, L.A.B. , Hadoux, X. & Wu, Z. (2022) Exploring reticular Pseudodrusen extent and impact on Mesopic visual sensitivity in intermediate age‐related macular degeneration. Investigative Ophthalmology & Visual Science, 63, 14. 10.1167/iovs.63.6.14 PMC9206392 35704305

[aos70151-bib-0033] Lad, E.M. , Fleckenstein, M. , Holz, F.G. , Shen, L. , Priore, L.V.D. , Silva, R. et al. (2024) Informing endpoints for clinical trials of geographic atrophy. Annual Review of Vision Science, 10, 455–476. 10.1146/annurev-vision-101922-045110 PMC12882265 39292553

[aos70151-bib-0034] Lindblad, A.S. , Lloyd, P.C. , Clemons, T.E. , Gensler, G.R. , Ferris, F.L. , Klein, M.L. et al. (2009) Change in area of geographic atrophy in the age‐related eye disease study: AREDS report number 26. Archives of Ophthalmology, 127, 1168–1174. 10.1001/archophthalmol.2009.198 PMC6500457 19752426

[aos70151-bib-0035] Marsiglia, M. , Boddu, S. , Bearelly, S. , Xu, L. , Breaux, B.E. , Freund, K.B. et al. (2013) Association between geographic atrophy progression and reticular Pseudodrusen in eyes with dry age‐related macular degeneration. Investigative Ophthalmology & Visual Science, 54, 7362. 10.1167/iovs.12-11073 PMC3823546 24114542

[aos70151-bib-0036] Martinez Villarruel Hinnerskov, J. , Krogh Nielsen, M. , Kai Thomsen, A. , Steffensen, M.A. , Honoré, B. , Vorum, H. et al. (2024) Chemokine receptor profile of T cells and progression rate of geographic atrophy secondary to age‐related macular degeneration. Investigative Ophthalmology & Visual Science, 65, 5. 10.1167/iovs.65.1.5 PMC10768715 38165703

[aos70151-bib-0037] Meleth, A.D. , Mettu, P. , Agrón, E. , Chew, E.Y. , Sadda, S.R. , Ferris, F.L. et al. (2011) Changes in retinal sensitivity in geographic atrophy progression as measured by microperimetry. Investigative Ophthalmology & Visual Science, 52, 1119–1126. 10.1167/iovs.10-6075 PMC3053096 20926818

[aos70151-bib-0038] Mokrane, A. , Zureik, A. , Bonnin, S. , Erginay, A. , Lavia, C. , Gaudric, A. et al. (2021) Retinal sensitivity correlates with the superficial vessel density and inner layer thickness in diabetic retinopathy. Investigative Ophthalmology & Visual Science, 62, 28. 10.1167/iovs.62.14.28 PMC8648065 34846517

[aos70151-bib-0039] Moussa, K. , Lee, J.Y. , Stinnett, S.S. & Jaffe, G.J. (2013) Spectral domain optical coherence tomography‐determined morphologic predictors of age‐related macular degeneration‐associated geographic atrophy progression. Retina, 33, 1590–1599. 10.1097/IAE.0b013e31828d6052 23538573

[aos70151-bib-0040] Nassisi, M. , Baghdasaryan, E. , Borrelli, E. , Ip, M. & Sadda, S.R. (2019) Choriocapillaris flow impairment surrounding geographic atrophy correlates with disease progression. PLoS One, 14, e0212563. 10.1371/journal.pone.0212563 PMC6386298 30794627

[aos70151-bib-0041] Niu, S. , de Sisternes, L. , Chen, Q. , Rubin, D.L. & Leng, T. (2016) Fully automated prediction of geographic atrophy growth using quantitative spectral‐domain optical coherence tomography biomarkers. Ophthalmology, 123, 1737–1750. 10.1016/j.ophtha.2016.04.042 27262765

[aos70151-bib-0042] Ouyang, Y. , Heussen, F.M. , Hariri, A. , Keane, P.A. & Sadda, S.R. (2013) Optical coherence tomography–based observation of the natural history of Drusenoid lesion in eyes with dry age‐related macular degeneration. Ophthalmology, 120, 2656–2665. 10.1016/j.ophtha.2013.05.029 PMC5340146 23830761

[aos70151-bib-0043] Pfau, M. , Künzel, S.H. , Pfau, K. , Schmitz‐Valckenberg, S. , Fleckenstein, M. & Holz, F.G. (2023) Multimodal imaging and deep learning in geographic atrophy secondary to age‐related macular degeneration. Acta Ophthalmologica, 101, 881–890. 10.1111/aos.15796 PMC11044135 37933610

[aos70151-bib-0044] Pilotto, E. , Guidolin, F. , Convento, E. , Spedicato, L. , Vujosevic, S. , Cavarzeran, F. et al. (2013) Fundus autofluorescence and microperimetry in progressing geographic atrophy secondary to age‐related macular degeneration. The British Journal of Ophthalmology, 97, 622–626. 10.1136/bjophthalmol-2012-302633 23410728

[aos70151-bib-0045] Querques, L. , Querques, G. , Forte, R. & Souied, E.H. (2012) Microperimetric correlations of autofluorescence and optical coherence tomography imaging in dry age‐related macular degeneration. American Journal of Ophthalmology, 153, 1110–1115. 10.1016/j.ajo.2011.11.002 22321805

[aos70151-bib-0046] Riley‐Gillis, B. , Huh, H. , Shen, J. & den Hollander, A.I. (2023) Genetic and molecular biomarkers for geographic atrophy. Acta Ophthalmologica, 101, 869–880. 10.1111/aos.15803 37933607

[aos70151-bib-0047] Sadda, S.R. , Guymer, R. , Holz, F.G. , Schmitz‐Valckenberg, S. , Curcio, C.A. , Bird, A.C. et al. (2018) Consensus definition for atrophy associated with age‐related macular degeneration on OCT. Ophthalmology, 125, 537–548. 10.1016/j.ophtha.2017.09.028 PMC11366072 29103793

[aos70151-bib-0048] Saeed, A. , Guymer, R.H. , Hadoux, X. , Jannaud, M. , Dang, D. , Hodgson, L.A.B. et al. (2025) Customized evaluation of progressive visual sensitivity loss in geographic atrophy to improve the power of clinical trials. Ophthalmology Science, 5, 100763. 10.1016/j.xops.2025.100763 PMC12018097 40276124

[aos70151-bib-0049] Sarici, K. , Abraham, J.R. , Sevgi, D.D. , Lunasco, L. , Srivastava, S.K. , Whitney, J. et al. (2022) Risk classification for progression to Subfoveal geographic atrophy in dry age‐related macular degeneration using machine learning–enabled outer retinal feature extraction. Ophthalmic Surgery, Lasers & Imaging Retina, 53, 31–39. 10.3928/23258160-20211210-01 34982004

[aos70151-bib-0050] Sayegh, R.G. , Kiss, C.G. , Simader, C. , Kroisamer, J. , Montuoro, A. , Mittermüller, T.J. et al. (2014) A systematic correlation of morphology and function using spectral domain optical coherence tomography and microperimetry in patients with geographic atrophy. The British Journal of Ophthalmology, 98, 1050–1055. 10.1136/bjophthalmol-2014-305195 24711655

[aos70151-bib-0051] Schmidt‐Erfurth, U. , Bogunovic, H. , Grechenig, C. , Bui, P. , Fabianska, M. , Waldstein, S. et al. (2020) Role of deep learning‐quantified Hyperreflective foci for the prediction of geographic atrophy progression. American Journal of Ophthalmology, 216, 257–270. 10.1016/j.ajo.2020.03.042 32277942

[aos70151-bib-0052] Schmitz‐Valckenberg, S. , Fleckenstein, M. , Göbel, A.P. , Hohman, T.C. & Holz, F.G. (2011) Optical coherence tomography and autofluorescence findings in areas with geographic atrophy due to age‐related macular degeneration. Investigative Ophthalmology & Visual Science, 52, 1–6. 10.1167/iovs.10-5619 20688734

[aos70151-bib-0053] Shen, L.L. , Sun, M. , Ahluwalia, A. , Young, B.K. , Park, M.M. & Del Priore, L.V. (2021) Geographic atrophy growth is strongly related to lesion perimeter: unifying effects of lesion area, number, and circularity on growth. Ophthalmol Retina, 5, 868–878. 10.1016/j.oret.2020.12.002 33307218

[aos70151-bib-0054] Shen, L.L. , Sun, M. , Grossetta Nardini, H.K. & Del Priore, L.V. (2020) Progression of Unifocal versus multifocal geographic atrophy in age‐related macular degeneration. Ophthalmology Retina, 4, 899–910. 10.1016/j.oret.2020.03.020 PMC7483721 32423772

[aos70151-bib-0055] Sunness, J.S. , Rubin, G.S. , Zuckerbrod, A. & Applegate, C.A. (2008) Foveal‐sparing scotomas in advanced dry age‐related macular degeneration. Journal of Visual Impairment & Blindness, 102, 600–610. PMC2836024 20224750

[aos70151-bib-0056] Takahashi, A. , Ooto, S. , Yamashiro, K. , Oishi, A. , Tamura, H. , Nakanishi, H. et al. (2016) Photoreceptor damage and reduction of retinal sensitivity surrounding geographic atrophy in age‐related macular degeneration. American Journal of Ophthalmology, 168, 260–268. 10.1016/j.ajo.2016.06.006 27296489

[aos70151-bib-0057] Thomsen, A.K. , Gøttsche, L.F. , Hinnerskov, J.M.V. , Falk, M.K. & Sørensen, T.L. (2024) Microperimetry and structural risk factors on OCT in intermediate age‐related macular degeneration. Ophthalmology Retina, 8, 786–793. 10.1016/j.oret.2024.02.016 38442827

[aos70151-bib-0058] Wong, E.N. , Chew, A.L. , Morgan, W.H. , Patel, P.J. & Chen, F.K. (2017) The use of Microperimetry to detect functional progression in non‐Neovascular age‐related macular degeneration: a systematic review. Asia‐Pacific Journal of Ophthalmology, 6, 70–79. 10.22608/APO.201643 28161925

[aos70151-bib-0059] Wu, Z. , Ayton, L.N. , Luu, C.D. & Guymer, R.H. (2014) Relationship between retinal microstructures on optical coherence tomography and Microperimetry in age‐related macular degeneration. Ophthalmology, 121, 1445–1452. 10.1016/j.ophtha.2014.01.025 24629618

[aos70151-bib-0060] Wu, Z. , Hadoux, X. , Jannaud, M. , Glover, E.K. , Gee, E.E. , Hodgson, L.A.B. et al. (2024) Visual sensitivity loss in geographic atrophy: structure‐function evaluation using defect‐mapping Microperimetry. Investigative Ophthalmology & Visual Science, 65, 36. 10.1167/iovs.65.1.36 PMC10807497 38241029

[aos70151-bib-0061] Wu, Z. , Hadoux, X. , Jannaud, M. , Martin, K.R. , Van Wijngaarden, P. & Guymer, R.H. (2023) Systematic underestimation of visual sensitivity loss on Microperimetry: implications for testing protocols in clinical trials. Translational Vision Science & Technology, 12, 11. 10.1167/tvst.12.7.11 PMC10341291 37428130

[aos70151-bib-0062] Wu, Z. , Luu, C.D. , Ayton, L.N. , Goh, J.K. , Lucci, L.M. , Hubbard, W.C. et al. (2015) Fundus autofluorescence characteristics of nascent geographic atrophy in age‐related macular degeneration. Investigative Ophthalmology & Visual Science, 56, 1546–1552. 10.1167/iovs.14-16211 PMC4349109 25678689

[aos70151-bib-0063] Wu, Z. , Steffen, V. , Harris, W. , Cukras, C.A. , Ferrara, D. & Guymer, R.H. (2025) Reading performance in geographic atrophy: comparison of different Reading speed measures for capturing longitudinal changes. Ophthalmology Science, 5, 100700. 10.1016/j.xops.2024.100700 PMC11930067 40124312

[aos70151-bib-0064] Yehoshua, Z. , de Amorim Garcia Filho, C.A. , Nunes, R.P. et al. (2014) Systemic complement inhibition with eculizumab for geographic atrophy in age‐related macular degeneration: the COMPLETE study. Ophthalmology, 121, 693–701. 10.1016/j.ophtha.2013.09.044 PMC4015213 24289920

[aos70151-bib-0065] Yehoshua, Z. , Rosenfeld, P.J. , Gregori, G. , Feuer, W.J. , Falcão, M. , Lujan, B.J. et al. (2011) Progression of geographic atrophy in age‐related macular degeneration imaged with spectral domain optical coherence tomography. Ophthalmology, 118, 679–686. 10.1016/j.ophtha.2010.08.018 PMC3070862 21035861

[aos70151-bib-0066] You, Q.S. , Camino, A. , Wang, J. , Guo, Y. , Flaxel, C.J. , Hwang, T.S. et al. (2021) Geographic atrophy progression is associated with Choriocapillaris flow deficits measured with optical coherence tomographic angiography. Investigative Ophthalmology & Visual Science, 62, 28. 10.1167/iovs.62.15.28 PMC8727307 34964802

