# Supplementary material for: Junctional sensitivity and atrophy progression rate of geographic atrophy secondary to age‐related macular degeneration
Source: Acta Ophthalmol. 2026 Apr 18;104(8):917–25. doi: 10.1111/aos.70151 (PMC13751859; doi:10.1111/aos.70151)
Supplement: Supplementary file 1 [file AOS-104-917-s001.pdf]

Supplementary table 2. Association between atrophy progression rate and baseline mean junctional sensitivity stratified by fundus autofluorescence (FAF) pattern adjusted for age.

FAF pattern	Beta Coefficient (95% CI) [µm/year/dB]	*P* value
None (n = 7)	-17.70 (-43.34 to 7.96)	0.13
Banded (n = 7)	2.01 (-22.93 to 27.06)	0.83
Focal (n = 3)	-16.20*	NA*
Patchy (n = 6)	-25.76 (-183.00 to 131.49)	0.64
Diffuse non-trickling (n = 24)	5.24 (-12.03 to 22.52)	0.54
Diffuse trickling (n = 9)	-6.31 (-42.96 to -1.28)	**0.041**

Bold values indicate statistical significance ($P < 0.05$).
CI = confidence interval.
*Insufficient sample size to compute 95% CI and *P* value.
