# Supplementary material for: Junctional sensitivity and atrophy progression rate of geographic atrophy secondary to age‐related macular degeneration
Source: Acta Ophthalmol. 2026 Apr 18;104(8):917–25. doi: 10.1111/aos.70151 (PMC13751859; doi:10.1111/aos.70151)
Supplement: Supplementary file 2 [file AOS-104-917-s003.pdf]

Supplementary table 1. Association between atrophy progression rate and baseline microperimetric retinal sensitivity.

	**Multivariable linear regression***	
	Beta Coefficient (95% CI) [µm/year]	*P* value
Mean junctional sensitivity [dB]	-7.89 (-18.15 to 2.38)	0.12
Junctional intraretinal hyperreflective foci	3.53 (-90.71 to 97.77)	0.94
Junctional subretinal drusenoid deposits	-15.93 (-131.71 to 99.85)	0.78
Baseline atrophy area [mm^2]	0.37 (-7.21 to 7.95)	0.92
Lesion focality		
Unifocal	Ref.	
Multifocal	92.51 (-4.89 to 189.90)	0.062

CI = confidence interval.
*Multiple regression adjusted for age, presence of junctional intraretinal hyperreflective foci, subretinal drusenoid deposits, baseline atrophy area, and lesion focality.
